# Single-site multiport vs. conventional multiport robot-assisted radical prostatectomy: A propensity score matching comparative study

**DOI:** 10.3389/fsurg.2022.960605

**Published:** 2022-09-28

**Authors:** Weibin Hou, Bingzhi Wang, Lei Zhou, Lan Li, Chao Li, Peng Yuan, Wei Ouyang, Hanyu Yao, Jin Huang, Kun Yao, Long Wang

**Affiliations:** ^1^ Department of Urology, the Third Xiangya Hospital, Central South University, Changsha, China; ^2^ Department of Urology, Ningxiang Hospital Affiliated to Hunan University of Traditional Chinese Medicine, Changsha, China

**Keywords:** robotic-assisted radical prostatectomy, single-site surgery, prostate cancer, same day discharge, extraperitoneal approach

## Abstract

**Objective:**

Robot-assisted radical prostatectomy (RARP) is a dynamically evolving technique with its new evolution of single-site RARP. Here we sought to describe our extraperitoneal technique, named the single-site multiport RARP (ssmpRARP) using the da Vinci Si^®^ platform and compare it with the transperitoneal conventional multiport RARP (cmpRARP).

**Materials and Methods:**

Data were retrospectively collected for patients who underwent RARP for localized prostate cancer from June 2020 to January 2022 in a single center. Propensity score matching was performed based on age, prostate size, body mass index, neoadjuvant hormonal therapy usage, prostate-specific antigen levels, and clinical T stage. The differences between the matched two groups were investigated.

**Results:**

Of the patients, 20 underwent ssmpRARP and 42 underwent cmpRARP during the period. After matching, 18 patients from each group were selected. Median follow-up was 7.8 months (2–12 months) for the ssmpRARP group, and 15.0 months (3–26 months) for cmpRARP. The demographic features between the two groups were comparable. The median total operative time, estimated blood loss, pathologic data, early follow-up outcomes, and hospitalization stays and costs were similar between the two groups. The ssmpRARP group tended to return to their bowel activities earlier (44.78 ± 10.83 h vs. 54.89 ± 12.97 h, *p* = 0.016). There were no significant differences in complication rates.

**Conclusions:**

We demonstrated the feasibility and safety of performing extraperitoneal ssmpRARP using the da Vinci Si^®^ robotic platform. Our technique showed comparable short-term outcomes with the transperitoneal cmpRARP. Prospective trials and long-term follow-up are necessary to confirm these results.

## Introduction

Radical prostatectomy is now the cornerstone in the treatment of moderate-risk and high-risk localized prostate cancer with a curative aim. It is also an appropriate first step in a multidisciplinary approach for men with locally advanced prostate cancer or even a low-volume metastatic disease (1–3). Robot-assisted radical prostatectomy (RARP) has now dominated the field of radical prostatectomy in developed western countries due to its obvious perioperative advantages (4). Recent years have witnessed an evolution of RARP techniques to be less invasive, provide better cosmetics, and have quicker postoperational recovery. The advent of next-generation robotic machines like the da Vinci SP® surgical system (Intuitive Surgical, Sunnyvale, CA, United States) has accelerated this evolution. At the same time, outpatient RARP and same-day-discharge (SDD) RARP have been proposed and performed more and more widely (5). Single-port RARP by da Vinci SP® surgical system has been demonstrated to be associated with a shorter hospital stay as well as less postoperative pain (6). Different techniques based on the da Vinci SP® surgical system have been confirmed to be safe and feasible, including transperitoneal (7), extraperitoneal (8), transvesical (9), and transperineal (10) single-port RARP. However, in many healthcare settings, the much higher costs of this next-generation refined robotic machine and disposable instruments decrease its availability and accessibility to patients in developing countries with limited healthcare budgets like China.

Several urology groups have tried single-port RARP using the conventional da Vinci Si® or Xi® surgical systems in China when the da Vinci SP® surgical system was unavailable. For instance, a group from Shanghai has described their initial experience of single-port transperitoneal (11) as well as extraperitoneal (12) RARP using the da Vinci Si® surgical system. Another group from Shanghai performed single-port transvesical RARP (13) and a group from Hangzhou described their initial experience of transperineal single-port RARP (14). All these groups have confirmed that single-port RARP with the conventional da Vinci Si® or Xi® surgical systems is safe and feasible, and could achieve most of the RARP techniques including anatomic nerve-sparing technique (15). One step further, a group from Chengdu has reported their experience of extraperitoneal single-site RARP without dedicated access device (16–18). Their modified technique enables surgeons from hospitals without any commercial multichannel port devices to perform single-site RARP with the da Vinci Si® or Xi® surgical systems, and at the same time reduce the cost of surgery significantly. Due to its multiport nature, we named is as single-site multiport robot-assisted radical prostatectomy (ssmpRARP). In the present paper, we sought to describe our extraperitoneal ssmpRARP technique and compare it with the transperitoneal conventional multiport RARP (cmpRARP).

## Materials and methods

### Patient selection

A total of 78 consecutive patients with biopsy-confirmed PCa who underwent RARP by a single surgeon using the da Vinci Si® system (Intuitive Surgical, Sunnyvale, CA, United States) between June 2020 and January 2022 were identified. Patients were operated using transperitoneal cmpRARP or extraperitoneal ssmpRARP at the discretion of the surgeon. Sixteen patients were excluded from further analysis because they had oligometastatic disease or clinical T4 stage disease. As a result, 20 patients underwent ssmpRARP and 42 patients underwent cmpRARP were included for this study. All the patients are Chinese. The data were collected following institutional review board approval and informed consent signed by each individual. This study was performed in accordance with the ethical standards of the Declaration of Helsinki and its later amendments.

### Propensity score matching

To reduce bias, propensity score matching on 20 patients who underwent ssmpRARP were matched to patients (1:1) from a cohort of 42 who underwent cmpRARP. The covariates included in the propensity score were age at surgery, body mass index (BMI), prostate volume, prostate-specific antigen (PSA) levels, clinical T stage, and whether they underwent neoadjuvant hormonal therapy. These variables were selected based on the known influencing factors and potential confounders on surgical outcomes. Patients were then matched using multivariate logistic regression including all the covariates. The matching used the nearest-neighbor algorithm with a 1:1 ratio without replacement. Covariates with a standardized difference of <|0.02| were considered balanced. Logistic regression and matching were performed using IBM SPSS (version 26.0; IBM Corp., Armonk, NY, United States).

### Surgical techniques of ssmpRARP

Under general anesthesia, the patient was secured in a low lithotomy position. After appropriate cleaning and draping of the lower abdomen, external genitalia, and upper thigh, a Foley catheter is placed into the bladder for potential manipulation during the operation.

**Figure 1:**
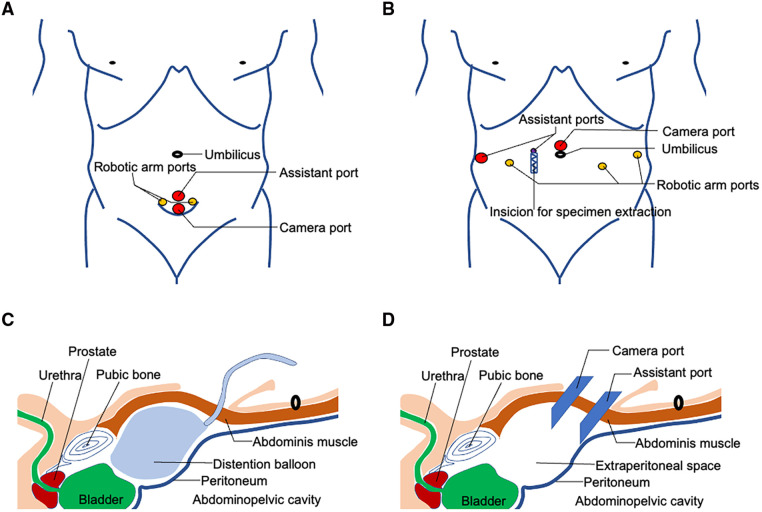
Schematic view of skin incision, extraperitoneal space creation, and port placement of ssmpRARP: (**A**) Trocar placement for ssmpRARP; (**B**) Six-port conventional multiport approach for RARP; (**C**) Extraperitoneal space creation using a distension balloon; (**D**) Extraperitoneal space access and trocar placement for ssmpRARP.

**Figure 2:**
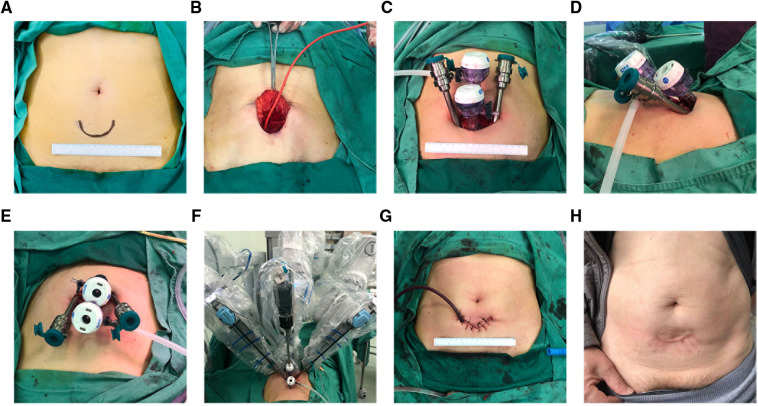
Operative view of skin incision, extraperitoneal space creation, and trocar placement and docking: (**A**) Marker of skin incision; (**B**) Balloon insufflation of the extraperitoneal space; (**C**) Four-port configuration with a caudocephalad view; (**D**) Four-port configuration with a lateral view; (**E**) Four-port configuration with a cephalocaudal view; (**F**) Docking configuration; (**G**) Wound closure and a drainage placement; (**H**) Appearance of abdominal incision 1 year after the surgery.

A single 5 cm curved incision with its arc toward crania is made three fingerbreadths above the pubic symphysis (Figure 1A). After the anterior rectus fascia is reached, blunt dissection is performed using the index finger between the subcutaneous adipose tissue and the anterior rectus fascia in order to create an initial space in between. Retracting the superior edge of the incision cranially, the inferior edge caudally, and side edges laterally, till their limits, a stab 1.5 cm incision is made on the anterior rectus fascia 2 cm below the umbilicus in the midline. Blunt separation of the rectus abdominis muscle is done until reaching the posterior rectus sheath. Blunt dissection using the index finger was performed above the posterior rectus fascia to create a preperitoneal working space. Here, a homemade space maker—an inflated surgical glove—or commercially available distension balloon could be introduced through the fascia incision to create the preperitoneal working space (Figure 1C). After the preperitoneal working space has been developed, a 12-mm regular trocar was inserted and the camera was introduced temporarily for the subsequent insertions of the inferior 12 mm da Vinci trocar, 4 cm midline below the first one. This inferior trocar was the actual optic trocar during operation (Figures 1A,D). The left lateral and right lateral da Vinci 8 mm trocars were inserted at the middle level of the first two trocars on the margin of rectus abdominis under direct visualization, one on each side (Figure 1A). The lateral da Vinci trocars were the surgeon's working ports while the superior regular 12 mm trocar was for the assistant (Figures 1A,D). Figures 2A–F show important steps during skin cutting, port placement, and docking in ssmpRARP.

**Figure 3:**
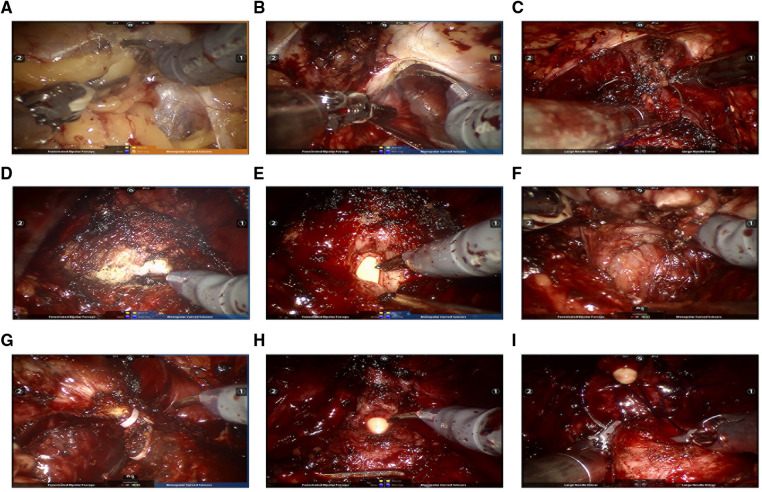
Intraoperative images illustrating key steps for ssmpRARP: (**A**) Creating an extraperitoneal working space; (**B**) Incising pelvic fascia of both sides; (**C**) Ligating and transecting the dorsal vascular complex; (**D**) Bladder neck dissection; (**E**) Dissecting the prostatic base and transecting the bladder neck; (**F**) Dissecting seminal vesicles and the posterior prostatic plane (Denonvilliers fascia). (**G**) Dissecting the prostatic pedicle (right); (**H**) Dissecting the apex of the prostate and transecting the urethral; (**I**) Performing vesicourethral anastomosis.

Then the patient was placed in a 15°–20° lithotomy Trendelenburg position. A 30° camera was installed looking upward during the surgery. The anterior prostatic fat was removed to create an extraperitoneal space (Figure 3A), and an incision of the lateral endopelvic fascia was followed (Figure 3B). After cutting off the pubovesical/puboprostatic ligaments, the dorsal venous complex was ligated using an absorbable suture (Figure 3C). Then, the bladder neck was identified and dissected (Figure 3D). The seminal vesicles and the vasa deferentia were exposed after transecting the bladder neck (Figure 3E). The vasa deferentia were transected at the ends and their distal part was used for suspending the prostate to expose the Denonvilliers fascia using sharp dissection (Figure 3F). Next, the prostate was dissected posteriorly from the Denonvilliers fascia until the neurovascular bundle (NVB) being identified. Releasing the NVB from the posterolateral prostatic surface by transecting the lateral vascular pedicles with the help of Hemolok clipping (Figure 3G). The dissection is carried out toward the prostatic apex. After transecting the distal urethra at the level of the urethroprostatic junction in a sharp and direct way (Figure 3H), the prostate was removed entirely and put aside. Limited lymph node dissection (obturator lymph nodes) was performed bilaterally after the prostate being out of the way, if necessary. Urethrovesical anastomosis was then performed in a continuous suturing way to reestablish the continuity of the urinary tract (Figure 3I). The integrity of urethrovesical anastomosis was routinely checked by an intraoperative flush test using about 150 ml saline through the catheter. Additional sutures were performed when needed. Finally, the specimens are removed through a sample bag, and a postoperative pelvic drain was placed in the same incision before wound closure (Figure 2G).

### Surgical techniques of cmpRARP

CmpRARP was performed using a six-port transperitoneal approach with the da Vinci Si® surgical system (Figure 1B). Under general anesthesia, the patient was placed in a 65°–70° lithotomy Trendelenburg position with legs apart. Operative steps were similar as described (19).

### Preoperative, operative, and postoperative data and statistics

The preoperative evaluation and postoperative care protocols did not differ between the two groups. Postoperative ambulation was encouraged, postoperative pain was controlled mainly by nonsteroidal analgesics, and the Foley catheter was removed 2 weeks postoperatively. Data on demographic characteristics (e.g., age, BMI, preoperative PSA, prostate volume, clinical T stages, rate of neoadjuvant hormonal therapy, and D’Amico risk groups), perioperative data [e.g. operation time, estimated blood loss (EBL), perioperative complications, and return time of bowel sounds], pathologic data [e.g., pathologic stage, lymph node dissection, positive surgical margins (PSMs)], early follow-up outcomes (e.g., 4 weeks after catheter removal continence and 6-week postoperative PSA), and hospitalization costs were collected and analyzed. Continent was defined as no pad or a single secure pad daily. PSA persistence was defined as a PSA ≥ 0.1 ng/ml 6 weeks after radical surgery. Biochemical recurrence (BCR) was diagnosed when it was >0.4 ng/ml after being undetectable at 6 weeks postoperatively. Parameters and outcomes were analyzed using the *t*-test, chi-square test, and Fisher's exact test using IBM SPSS 26.

## Results

### Patient demographics

Median follow-up was 7.8 months (2–12 months) for the ssmpRARP group and 15.0 months (3–26 months) for the cmpRARP group. Both groups showed similar demographics and no significant differences in clinical parameters after propensity score matching [mean age (years), 67.40 ± 6.56 vs. 65.49 ± 8.04, *p* = 0.4; mean BMI (kg/m^2^), 23.83 ± 2.93 vs. 23.97 ± 2.67, *p* = 0.9; mean PSA level (ng/ml), 21.64 ± 22.73 vs. 21.22 ± 19.41, *p* = 1.0; mean prostate volume (ml), 31.38 ± 12.13 vs. 34.22 ± 11.63, *p* = 0.5]. The distribution of patients among the clinical T stages was balanced (cT1: 5.6% vs. 11.1%, cT2: 83.3% vs. 77.8%, cT3: 11.1% vs. 11.1%); the D’Amico risk groups was balanced between the two groups (low: 5.6% vs. 5.6%, median: 38.9% vs. 33.3%, high: 55.6% vs. 61.1%) (detailed in Table 1).

**Table 1:** Demographics and preoperative characteristics.

Parameter	ssmpRARP	cmpRARP	*p*-value
Age (years)	67.40 ± 6.56	65.49 ± 8.04	0.4
BMI (kg/m^2^)	23.83 ± 2.93	23.97 ± 2.67	0.9
Prostate volume (ml)	31.38 ± 12.13	34.22 ± 11.63	0.5
PSA level (ng/ml)	21.64 ± 22.73	21.22 ± 19.41	1.0
Clinical T stage [*n* (%)]			
cT1	1 (5.6)	2 (11.1)	
cT2	15 (83.3)	14 (77.8)	
cT3	2 (11.1)	2 (11.1)	0.8
Risk group [*n* (%)]			
Low risk	1 (5.6)	1 (5.6)	
Moderate risk	7 (38.9)	6 (33.3)	
High risk	10 (55.6)	11 (61.1)	0.9
Neoadjuvant hormonal therapy [*n* (%)]	3 (16.7)	2 (11.1)	0.5

ssmpRARP, single-site multiport robot-assisted radical prostatectomy; cmpRARP, conventional multiport robot-assisted radical prostatectomy; BMI, body mass index; PSA, prostate-specific antigen.

### Perioperative outcomes

No significant differences were noted between the ssmpRARP and cmpRARP groups in terms of operation time (209.17 ± 68.34 vs. 230.00 ± 68.81 min, *p* = 0.4) and EBL (180.56 ± 184.02 vs. 211.11 ± 117.02 min, *p* = 0.6). Of the 18 patients who underwent ssmpRARP and 18 who underwent cmpRARP, 11 (61.1%) had pT2 tumors in both groups and the rest (38.9%) had pT3 tumors. A similar proportion of patients underwent lymph node dissection in both groups (ssmpRARP vs. cmpRARP: 44.4% vs. 55.6%, *p* = 0.8). Among those who received lymph node dissection, all those in the cmpRARP group received an extended pelvic lymph node dissection while all those in the ssmpRARP group received a limited one. The number of lymph nodes removed was significantly less in the ssmpRARP group (3.5 vs. 9, *p* = 0.003). PSMs showed no significant difference (*p* = 0.7): six patients (33.3%) had PSM in the ssmpRARP group and seven (38.9%) in the cmpRARP group. The patients who underwent ssmpRARP tend to return to their bowel activities earlier (44.78 ± 10.83 h vs. 54.89 ± 12.97 h, *p* = 0.016), potentially due to its extraperitoneal approach. The total length of stay was comparable in both groups (11.78 ± 4.52 vs. 12.33 ± 2.93 days, *p* = 0.7) and median day of discharge after surgery was day 5 for the ssmpRARP group and day 6 for the cmpRARP group.

PSA persistence, which is defined as a PSA level ≥0.1 ng/ml 6 weeks after radical surgery, was identified for two patients in the ssmpRARP group and five patients in the cmpRARP group. The functional outcome in the form of urinary continence at 4 weeks after catheter removal (6 weeks after surgery) was compared in both groups (44.4% in ssmpRARP vs. 50.0% in cmpRARP group, *p* = 0.7). A total of four cases reported surgical complications of Clavien–Dindo classification of ≥2. Among them, one intraoperative blood transfusion has been described in the ssmpRARP group; one patient from the cmpRARP group developed deep vein thrombosis that needed a full anticoagulation therapy; both groups had one case of orchiepididymitis while with a Foley catheter, they got an antibiotic treatment. The total cost between the two groups were comparable (81,448.10 ± 11,075.95 for the ssmpRARP group vs. 84,975.86 ± 5,730.83 for the cmpRARP group) (detailed in Table 2). One example of the appearance of skin incision scar at 1 year after surgery is shown in Figure 2H. The relatively small patient sample size and short follow-up period limit interpretation of overall and BCR survival outcomes.

**Table 2:** Perioperative, pathologic, and early follow-up data.

Parameter	ssmpRARP	cmpRARP	*p*-value
Operation time (min)	209.17 ± 68.30	230.33 ± 68.81	0.4
Estimated blood loss (ml)	180.56 ± 184.02	211.11 ± 117.02	0.6
Pathologic T stage [*n* (%)]			
pT2	11 (61.1)	11 (61.1)	
pT3	7 (38.9)	7 (38.9)	1.0
Gleason score			
<7	1	3	
7	12	10	
>7	5	5	0.6
Lymph node dissection [*n* (%)]	8 (44.4)	9 (55.6)	0.8
No. of lymph node removed [median (IQR)]	3.5 (2–4.75)	9 (7–12)	**0**.**003**
Positive rate of lymph node removed	10.0%	12.0%	0.8
Positive surgical margin [*n* (%)]	6 (33.3)	7 (38.9)	0.7
Positive surgical margin in cT2 [*n* (%)]	4 (26.7)	6 (42.9)	0.1
Postoperative complications [*n* (%)]	2 (11.1)	2 (11.1)	0.2
Time to return of bowel sounds (h)	44.78 ± 10.83	54.89 ± 12.97	**0**.**016**
Total length of stay (days)	11.78 ± 4.52	12.33 ± 2.93	0.7
Stay after surgery (days)	4.83 ± 2.12	5.94 ± 2.15	0.2
Zero PSA at 6 weeks [*n* (%)]	16 (88.9)	13 (72.2)	0.2
Continence at 1 month [*n* (%)]	8 (44.4)	9 (50.0)	0.7
Total cost [Yuan (￥)]	81,448.10 ± 11,075.95	84,975.86 ± 5,730.83	0.2

ssmpRARP, single-site multiport robot-assisted radical prostatectomy; cmpRARP, conventional multiport robot-assisted radical prostatectomy; PSA, prostate-specific antigen.

Bold values of *p*-value are less than 0.05, which means statistically significant.

## Discussion

Radical prostatectomy is increasingly performed to treat patients with prostate cancer (20), with the robot-assisted approach accounting for most of the procedures in the United States (21), Europe (22), and some first echelon hospitals in China (23). RARP has revolutionized the surgical removal of prostate by providing great ergonomics, versatile dexterity, and immersive three-dimensional visual field for surgeons. However, RARP has long been criticized for high equipment costs and disposable materials (24), as well as only minimal if any oncological or functional benefits over open radical prostatectomy (25, 26). It should be emphasized that robotic surgical equipment and techniques have continued to evolve during the years since its origin. A variety of novel robotic techniques for radical prostatectomy have been introduced to optimize oncological and functional outcomes (27), most of which would be unfeasible or technically challenging otherwise. One of the most promising techniques is the single-port/single-site RARP, usually based on the da Vinci SP® robot platform, which is gaining traction worldwide. Here we described our refined single-site RARP technique based on the conventional da Vinci Si® or Xi® surgical systems, ssmpRARP. In our early experience, we were able to complete all 20 surgeries with acceptable operative time, minimal blood loss, and no increased intra- or postoperative complications. Compared with cmpRARP, ssmpRARP could achieve noninferior outcomes with respect to surgical margins, postoperative PSA detection, operation time, and estimated blood loss. Although single-site surgery using the da Vinci SP® robot platform definitely would have advantages over that based on the da Vinci multiarm robotic system (28), our experience indicates that ssmpRARP might serve as an example of providing the most advanced surgical cancer care when the availability of health budget, hospital beds, and inpatient staffing are strained or even shrank especially during the coronavirus 2019 (COVID-19) pandemic in developing countries like China.

Our current ssmpRARP was performed without any special channel devices. Although commercially available multichannel port device could ease the initiation of surgery and allow a tighter seal to maintain pneumoperitoneum, it has some drawbacks. First, to create a better device–skin attachment and a gas-tight seal, the outer as well as inner rings of the device have to be relatively thick, which would increase the depth of the instruments into the abdominal cavity and limit their maneuverability. On the other hand, insertion of a multichannel port requires a full thickness incision of the sheath of rectus abdominis, increasing the risk of visceral trauma and postsite hernia (12). Single-site RARP without special channel device would avoid some of these drawbacks (17). Endoscopic instruments might be potentially more flexible, and three of four small independent port-size incisions on the inner sheath of rectus abdominis would lower the risk of postsite hernia and postsurgery pain. Wang's study comparing single-incision RARP with and without an extraperitoneal special channel device showed that single-incision RARP without an extraperitoneal special channel device is safe and feasible, and costs less than that with a special channel device (17).

The ssmpRARP technique has several advantages. First, the single-port/single-site RARP tips the balance in favor of extraperitoneal radical prostatectomy. The transperitoneal radical prostatectomy would violate the peritoneal cavity, resulting in adhesions, delayed bowel function return, and, even worse, bowel-related complications. The cmpRARP is usually performed transperitoneally since three or even four robotic arms require more space to avoid conflicts outside or inside the abdomen (29). Extraperitoneal RARP, though being technically more difficult, could offer shorter operative time, faster discharge time, and less operative pain, at the same time with equivalent oncological outcomes and complication rates (30). In reality, the comfort of a large working space and improved visibility still make the transperitoneal approach favorable for most urologists (30). Single-port RARP could be performed in a smaller operating space, which allows it to be performed in extraperitoneal ways more easily (31). Compared with transperitoneal single-port RARP, extraperitoneal single-port RARP is associated with a significantly shorter postoperative hospital stay and decreased need for postoperative narcotics (32).

Second, ssmpRARP might be one of the cheapest modified ways for performing single-port/single-site RARP. RARP is associated with much higher costs than laparoscopic and open radical prostatectomy (33). In addition, the type of robotic platform also makes a difference, with the cost of the da Vinci® SP platform being significantly higher than that of the da Vinci® Xi platform, mostly due to the increased cost of instruments and consumables (34). Our ssmpRARP technique could be achieved using the conventional da Vinci Si® or Xi® surgical systems, which cost less than the da Vinci SP system (34). Recently, exquisite nerve-sparing techniques have been described using single-port RARP based on the da Vinci Si® platform, indicating its potential capacity for performing even the most advanced RARP techniques (15). On the other hand, our current ssmpRARP technique was performed without any special channel devices, reducing the cost further (17).

In addition, ssmpRARP, as a type of RARP technique, could be cost-effective compared with laparoscopic and open radical prostatectomy after accounting for better outcomes, decreased hospital stays, and low readmission rates. Recent evidence suggests that RARP is associated with fewer acute and chronic postoperative complications (35). When 1-year postdischarge healthcare cost was included for analysis, the higher cost during the index hospitalization of RARP compared with open radical prostatectomy could be balanced (36). If an over 3-year or even 10-year time horizon was used, RARP might be cost-saving due to reasons including lower complication rate and faster return to work (37, 38). Thus, higher costs of robotic prostatectomy may be offset by the long-term health gain. In conclusion, albeit associated with significant upfront expenditure, when one considers cumulative long-term healthcare costs, including the management of postoperative complications and functional outcomes, RARP, particularly ssmpRARP, could be cost-effective.

To be of significant importance, single-site RARP has been leveraged to widen the practice of SDD due to its obvious advantages of less postoperative pain and earlier convalescence. Single-site RARP causes a 15% reduction of pains compared with its multisite counterpart and allows 33% more patients opting for SDD (39). The adoption of SDD will offer significant cost savings to the healthcare system and expedite the recovery process of patients (40, 41). Moreover, SDD allows more provision of surgical cancer care when the availability of hospital beds and inpatient staffing are strained. Beyond accruing data on feasibility, new challenges presented by the COVID-19 pandemic further strengthened the argument for SDD after RARP. SDD after RARP, which is usually an elective procedure, would reserve more healthcare resources for combating COVID-19 and at the same time reduce the risk of nosocomial infection among hospital facilities and inpatients. Despite enthusiasm for facilitating SDD after RARP around the world during the past few years, SDD RARP remains infrequently used in routine practice (42). The situation in China is more disappointed. Hospitalization of 4–5 days remains a routine practice for Chinese RARP patients. The SDD mode of RARP in China is still in its infancy with only a few attempts making for the overnight RARP in highly selected patients (43). We attribute this to a lack of medical education among most Chinese patients and a lack of service capability of the community healthcare system in China. In addition, it is difficult for Chinese urologists to withstand the potential medical disputes caused by SDD. Thus, it is difficult to implement SDD in China at the present stage. However, this ssmpRARP technique represents our initial efforts to enhance the adoption of SDD after RARP in China.

Obviously ssmpRARP has some drawbacks. The use of a pure single-port approach limits the assistant bed participation during surgery and thus put more pressure on the surgeon. Although limited lymph node dissection is possible, extended pelvic lymph node dissection is difficult with the current setup. Therefore, we suggest that this technique is more appropriate for a specific cohort of patients with less possibility of lymph node metastasis. Performing ssmpRARP in patients with a large prostate could be potentially difficult since the size of the prostate could be a limitation for deployment, insertion, and exchange of the surgical arms. Previous infraumbilical midline incisions are the main contraindications because this could create a fibrous tissue that could make impossible the creation of the extraperitoneal space. Our study included only a small sample size with short follow-up, and it is too early to make definite comparisons between this new technique with the traditional ones.

Based on our initial series, single-site multiple port robot-assisted radical prostatectomy is a safe and feasible surgical approach. The propensity matched comparative study showed that this novel approach allows for similar surgical outcomes and costs but with fewer scars, and potentially increases the rate of SDD RARP. ssmpRARP serves as an example of our efforts to make a difficult procedure and a most advanced surgical technique accessible to a broader range of clinicians and patients, especially in this COVID-19 pandemic era and in potential future global crises. Larger series with longer follow-ups are required to further evaluate the practical advantages of the approach.

## Data Availability

The raw data supporting the conclusions of this article will be made available by the authors, without undue reservation.
